# Comparison of Mathematical Methods for Compensating a Current Signal under Current Transformers Saturation Conditions

**DOI:** 10.3390/s21217273

**Published:** 2021-11-01

**Authors:** Ismoil Odinaev, Aminjon Gulakhmadov, Pavel Murzin, Alexander Tavlintsev, Sergey Semenenko, Evgenii Kokorin, Murodbek Safaraliev, Xi Chen

**Affiliations:** 1 Department of Automated Electrical Systems, Ural Federal University, 620002 Yekaterinburg, Russia; ismoil.odinaev@urfu.ru (I.O.); p.v.murzin@urfu.ru (P.M.); a.s.tavlintsev@urfu.ru (A.T.); sergey.semenenko@urfu.ru (S.S.); murodbek_03@mail.ru (M.S.); 2 Research Center for Ecology and Environment of Central Asia, Xinjiang Institute of Ecology and Geography, Chinese Academy of Sciences, Urumqi 830011, China; aminjon@ms.xjb.ac.cn; 3 State Key Laboratory of Desert and Oasis Ecology, Xinjiang Institute of Ecology and Geography, Chinese Academy of Sciences, Urumqi 830011, China; 4 Ministry of Energy and Water Resources of the Republic of Tajikistan, Dushanbe 734064, Tajikistan; 5 STC JSC ”Federal Testing Center”, 734064 Yekaterinburg, Russia; evgenii.kokorin@gmail.com; 6 University of Chinese Academy of Sciences, Beijing 100049, China; 7 Sino-Belgian Joint Laboratory of Geo-Information, 9000 Ghent, Belgium; 8 Sino-Belgian Joint Laboratory of Geo-Information, Urumqi 830011, China

**Keywords:** current transformer saturation, initial flux density, curve magnetization, unsaturated section

## Abstract

Current measurements from electromagnetic current transformers are essential for the construction of secondary circuit systems, including for protection systems. Magnetic core of these transformers are at risk of saturation, as a result of which maloperation of protection algorithms can possibly occur. The paper considers methods for recovering a current signal in the saturation mode of current transformers. The advantages and disadvantages of methods for detecting the occurrence of current transformers core saturation are described. A comparative analysis of mathematical methods for recovering a current signal is given, their approbation was carried out, and the most promising of them was revealed. The stability and sensitivity of recovery methods were tested by adding white noise to the measured signal and taking into account the initial flux density (remanent magnetization) in the current transformers core. Their comparison is given on the basis of angular, magnitude, and total errors at a given simulation interval.

## 1. Introduction

One of the main measuring instruments in electric power facilities are electromagnetic current transformers (CT). As shown in [1], during short circuits (SC) these CT core could be saturated, whereby undesired operation of relay protection systems (RPS) may occur. In accordance with [2], CT saturation can affect the correct functioning, sensitivity, and response time of the RPS, as well as the fault location algorithms correct operation. All these factors ascertain the relevance of the CT saturation-conditioned errors compensation.

Two main approaches are used in order to reduce the CT measurements errors or to decrease its influence on RPS operation: a constructive change in the CT [3] and additional mathematical processing of signal [4,5,6,7,8,9,10,11,12,13,14,15,16,17,18,19,20,21,22,23,24,25,26,27,28,29,30].

The methods proposed in the first approach are based on the CT magnetic and electric circuits utilized [3]. The essence of these methods is to optimize the absolute magnetic permeability of the CT core $\nu_{a}$. One of these absolute permeability optimization methods is CT ratio correction [3]. Applying these techniques, it is possible to reduce CT measurement errors, but these methods work efficiently only when the primary current is in the range from 10% to 120% of nominal [3].

Within the second approach, in order to compensate for an error in the CT in its saturation mode or to exclude the influence of saturation on the operation of RPS, saturation detection [4,5,6,7,8,9,10] and compensation methods [11,12,13,14,15,16,17,18,19,20,21,22,23,24,25,26,27,28,29,30] are used. The latter, due to the complexity of adaptation to real conditions, have not found wide practical development. In terms of digital signal processing, the problem of the CT error compensating in saturation mode can be divided into three subtasks: segmentation, selection, and filtering. Segmentation is the distinction between normal and fault operating modes. Selection is the task of selecting the samples of the current signal that are in the zone of the unsaturated section (US), after which the CT saturates without the mode changing. Filtering is the task of restoring the distorted measured current signal samples in saturated section, or, in other words, the task of CT error compensation during saturation. Selection and filtering are the most important of all three listed subtasks. With reservations, one can say that the methods proposed within the second approach are designed to solve the problem of selection [4,5,6,7,8,9,10] and filtration [11,12,13,14,15,16,17,18,19,20,21,22,23,24,25,26,27,28,29,30].

The US selection methods, in turn, can be divided into two groups: selection using methods of mathematical analysis [4,5,6,7] and selection using methods of static data analysis [8,9,10]. The first group is based on the difference functions previously proposed in [18,31]. The advantages of these methods include the speed and relatively accurate determination of the last US sample. However, those methods are highly sensitive to white noise. The second group mainly determines the presence of saturation with the subsequent RPS blocking. The main advantage of the methods in this group is low sensitivity to white noise. However, with the help of these methods, it is not possible to accurately determine the last US sample, as a result the solution of the filtration problem becomes more complicated.

In the case of applying the filtering methods [11,12,13,14,15,16,17,18,19,20,21,22,23,24,25,26,27,28,29,30], the original “clean” signal is recovered by eliminating the component caused by the CT saturation. The essence of these methods is the use of CT parameters and measured current samples. Filtration methods were proposed after the appearance and active use of microprocessor devices in the electric power industry. These methods can be divided into following groups:

1. Compensation using magnetization curve [11,12,13,14];
2. Compensation by forecasting [15,16,17,18];
3. Compensation using neural networks [19,20,21,22,23,24,25];
4. Compensation using combined methods [26,27,28,29,30].

Within the paper, the analysis of filtration methods, their description, and testing are presented. In order to reveal the stability/sensitivity of these methods to white noise and the initial flux density $B_{0}$ values, white noise is superimposed in the model signal and $B_{0}$ is taken into account in the CT model. Advantages and disadvantages of methods are marked. The conclusions concerning the main properties of these methods are presented and the most promising of them are highlighted.

## 2. Methodology

### 2.1. Current Filtration Using Magnetization Curve

In [11,12], a method of filtering the current using the magnetization curve was proposed. For convenience, this method will further be designated as A1. The essence of this method is the use of $B=f\left(H\right)$, which allows to calculate the magnetizing current $i_{\mu }$. To obtain its value, the flux density *B* is used, which is calculated using the Equation (1).

(1) $$\begin{array}{cc} B(t)= & \frac{R_{2}}{w_{2}\cdot s}\cdot \int_{t_{0}}^{t}i_{2}\left(\lambda \right)d\lambda \\ \; & +\frac{L_{2}}{w_{2}\cdot s}\cdot \left(i_{2}\left(t\right)-i_{2}\left(t_{0}\right)\right)+B\left(t_{0}\right), \end{array}$$

where *s* is the cross-section of the CT core, m^2^; $R_{2}$ and $L_{2}$ are respectively the active resistance and inductance of the secondary CT circuit, Ohm and H; $i_{2}$ is the measured current, A; $w_{2}$ is the number of CT secondary winding turns.

Further, knowing the flux density, using dependence $B=f\left(H\right)$, one can determine the magnetic field strength *H* and, using Equation (2), calculate the magnetizing current $i_{\mu }$.

(2) $$i_{\mu }\left(t\right)=\frac{H(t)\cdot l}{w_{2}},$$

where *l* is the average length of the CT core magnetic path, m.

Next, using Equation (3), the filtered current is calculated.

(3) $$i_{1}^{\prime }\left(t\right)=i_{\mu }\left(t\right)+i_{2}\left(t\right)$$

In [13,14], a method for filtering the current based on the approach described in [11] is proposed. The first difference of [13,14] from [11] is the hysteresis loop considering, the second is considering of the magnetizing current components: eddy currents $i_{eddy}$ and hysteresis losses $i_{h}$.

Using Equation (4), the magnetizing current and its components are calculated.

(4) $$\left\{\begin{array}{cc} \frac{di_{h}}{dt} & =f\left(\mu_{0},B\left(t\right),\frac{dB}{di_{h}},\frac{dB}{dt}\right)\\ i_{eddy} & =f\left(\sigma ,\frac{dB}{dt}\right)\\ i_{\mu } & =i_{h}+i_{eddy} \end{array},\right.$$

where $\mu_{0}$ is the magnetic constant, σ is the eddy current loss factor.

The advantage of the proposed methods is robustness in the presence of noise and harmonic components in the measured current. However, the main disadvantage is high sensitivity to the unknown initial flux value in the CT core. The shape of the magnetization curve could condition a negative impact on the result of the method.

### 2.2. Filtering Current Using Forecasting Methods

In [15,16,17], methods of filtering the distorted measured CT current signal have been proposed. Those methods are based on the well-known least squares method (LSE). The paper [17] analyzes the methods of filtering the measured current of the CT by means of forecasting, where the LSE is presented as the most effective method. In [16], when the CT is saturated, the current is filtered using the LSE, and then it is passed to the input of the overcurrent protection. Thus, the correctness of the RPS operation in the CT saturation mode is assessed. This method will further be designated as A2.

In this model, the unknown parameters are: the magnitude *A* of the sinusoidal and the magnitude *B* of the exponential components, the decay rate of the exponential component λ and the initial phase of the short-circuit current φ, as seen in Equation (5).

(5) $$i_{1}^{\prime }\left(t\right)=A\cdot \sin\left(\omega \cdot t+\varphi \right)+B\cdot e^{-\lambda \cdot t},$$

where ω is the cyclic frequency, considered equal to the nominal frequency of the network (50 Hz).

The procedure for determining the main parameters of the input signal is as follows. Using the method of converting the sums and differences of the angles of trigonometric functions for the sine term, as well as the approximation of the first-order Taylor series for the exponential term, Equation (5) can be rewritten as follows:

(6) $$\begin{array}{cc} i_{1}^{\prime }\left(t\right)= & A\cdot \cos(\varphi )\cdot \sin(\omega \cdot t)\\ \; & +A\cdot \sin(\varphi )\cdot \cos(\omega \cdot t)-B\cdot \lambda \cdot t+B. \end{array}$$

Further, replacing the unknown parameters of Equation (6) with coefficients $C_{1}$–$C_{4}$, the equation will take the form (7).

(7) $$i_{1}^{\prime }\left(t\right)=C_{1}\cdot \sin\left(\omega \cdot t\right)+C_{2}\cdot \cos\left(\omega \cdot t\right)-C_{3}\cdot t+C_{4}$$

Based on the US samples (7) in matrix form, this Equation will be rewritten as:

(8) $$\underset{A}{\underset{︸}{\left(\begin{array}{cccc} \sin(\omega \cdot t_{0-1}) & \cos(\omega \cdot t_{0-1}) & 1 & t_{0-1}\\ \sin(\omega \cdot t_{0-2}) & \cos(\omega \cdot t_{0-2}) & 1 & t_{0-2}\\ \vdots & \vdots & \vdots & \vdots \\ \sin(\omega \cdot t_{0-n}) & \cos(\omega \cdot t_{0-n}) & 1 & t_{0-n} \end{array}\right)}}\times \underset{x}{\underset{︸}{\left(\begin{array}{c} C_{1}\\ C_{2}\\ C_{3}\\ C_{4} \end{array}\right)}}=\underset{b}{\underset{︸}{\left(\begin{array}{c} i_{1}^{\prime }\left(t_{0-1}\right)\\ i_{1}^{\prime }\left(t_{0-2}\right)\\ \vdots \\ i_{1}^{\prime }\left(t_{0-n}\right) \end{array}\right)}}$$

Indexes “0” and “*n*” in (8) are the numbers of samples corresponding to the beginning and end of US.

(9) $$\begin{array}{c} A\cdot x=b \end{array}$$

(10) $$\begin{array}{c} x={\left(A^{T}\cdot A\right)}^{-1}\cdot A^{T}\cdot b \end{array}$$

Thus, knowing the coefficients $C_{1}$–$C_{4}$ and substituting them into Equation (11), it is possible to predict the current in the saturation sections.

(11) $$i_{1}^{\prime }\left(t\right)=A_{calc}\cdot \sin\left(\omega \cdot t+\varphi_{calc}\right)+C_{4}\cdot e^{\frac{C_{3}}{C_{4}}\cdot t},$$

where $A_{calc}=\sqrt{C_{1}/C_{2}}$; $\varphi_{calc}=\arcsin\left(C_{2}/C_{1}\right)$; $C_{3}/C_{4}=-\left(\lambda \cdot B\right)/B=-\lambda$.

The advantage of the proposed method is high stability with respect to initial flux density in the CT core. However, the method has a high sensitivity to white noise and harmonics in the input measured current. In addition, the presence of noise has a significant effect on the accuracy of estimating the exponential component decay rate parameter.

**Figure 1:**
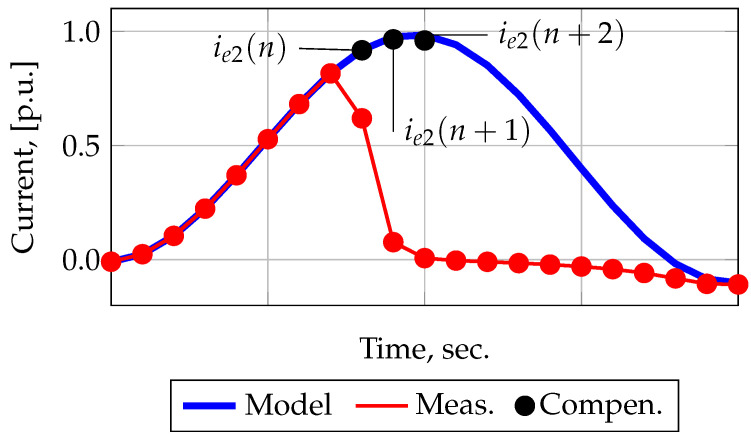
Artificial expansion of US.

The study [18] considers a method that allows US artificial expansion. This method will be designated as A3. The method is based on the use of the US measured current signal derivative, i.e., it is assumed that the increments of the secondary current in the US are constant. Thus, based on the US samples, the next sample outside this US can be predicted, as shown in Figure 1. Among the expressions proposed in [18], the third-order derivative has the highest accuracy, which is described in discrete form as follows:

(12) $$\begin{array}{cc} i_{2e}\left(n\right)= & 4\cdot i_{2}\left(n-1\right)-6\cdot i_{2}\left(n-2\right)\\ & \;+4\cdot i_{2}\left(n-3\right)-i_{2}\left(n-4\right). \end{array}$$

where *n* is the number of the first forecasted sample in the saturated section, $i_{2}$ is the measured samples of the secondary current, and $i_{2e}$ is the forecasted sample of the measured current signal obtained by extrapolation of US.

The advantages of the proposed method are: the absence of the need to use the parameters of the CT core and a small computational load of microprocessor devices. However, the proposed method is not capable of filtering the distorted current over the entire saturation interval, and its efficiency depends on the sampling frequency of the current signal.

### 2.3. Filtering Current Using Neural Networks

**Figure 2:**
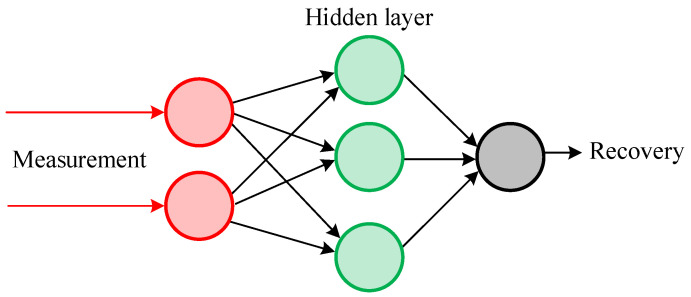
Example neural network.

In [19,20,21,22,23,24,25], methods for filtering the distorted measured current by training neural networks are proposed. The methods of neural networks considered in this paper are based mainly on the sigma function [32], located at the hidden level of the network, Figure 2 (filled neurons). Typically, the network topology is performed in the form of “feed-forward” and “feedback”. This method will further be designated as A4.

Equation (13) shows the dependence of the filtered current $i_{1}^{\prime }$ on the vector of the input distorted measured current signal $i_{2}$, on the weight coefficients $a_{ij}$, on the activation function $F_{k}$, and also on the adder *S* located at the output level of the network.

(13) $$i_{1}^{\prime }=F\left(i_{2},a_{ij},F_{k},S\right).$$

The advantages of the proposed methods are:

- There is no need to use CT parameters in the methods;
- Independence from US length;
- Methods are capable of filtering the current with high accuracy in different network modes and degrees of secondary current distortion.

However, taking into account the various network modes and the speed of modes change, the main disadvantage of the A4 method is the need to train and adapt them to all modes. Considering all the factors affecting the CT saturation, the latter leads to a significant increase in the requirements for the computational speed of microprocessor devices.

### 2.4. Filtering Current Using Combined Methods

In studies [26,27], an algorithm for filtering the distorted CT current in the saturation mode is proposed. The proposed method consists of two algorithms: finding the magnetizing current using an analytically preset magnetization curve and filtering the current using the A2 method.

To solve the filtration problem, a current model is used in the short circuit mode (5) with unknown coefficients $C_{1}$–$C_{4}$. The calculation of magnetizing current $i_{\mu }$ is shown in Equation (14).

(14) $$i_{\mu }=f\left(H\left(B\left(i_{2},C_{5}\right)\right)\right),$$

where $C_{5}$ is the unknown coefficient replacing the initial flux density $B\left(t_{0}\right)$ in the CT core. The expanded version of expression (14) is shown in (1)–(3).

In accordance with (5) and (14), to compensate distorted current, the vector function (15) is formulated:

(15) $$f\left(C\right)=i_{2}+f\left(i_{2},C_{5}\right)-A\cdot C_{1-4},$$

where $i_{2}$ is the vector of the secondary current measured values, C is the vector of unknown coefficients $C_{1}$,$C_{5}$, and A are the matrix based on the known terms on the right-hand side (5).

To search for the extremum of function (15), it is divided into two parts—(16) and (17).

(16) $$\begin{array}{c} C_{1-4}=A^{+}\cdot \left(i_{2}+f\left(i_{2},C_{5}\right)\right), \end{array}$$

(17) $$\begin{array}{c} f\left(C_{5}\right)=\left(E-A\cdot A^{+}\right)\cdot (i_{2}+f\left(i_{2},C_{5}\right)), \end{array}$$

where E is the identity matrix, $A^{+}$ is the matrix A pseudoinverse. In order to find the function extremum (17), the $C_{5}$ is determined, then $C_{1-4}$ is calculated by expression (16).

Thus, knowing the coefficients $C_{1-4}$, one can filter the current by forecasting. The advantage of this method is the high resistance to noise in the distorted current. The main disadvantage is the iterative nature of the procedure to obtain unknown coefficients.

**Figure 3:**
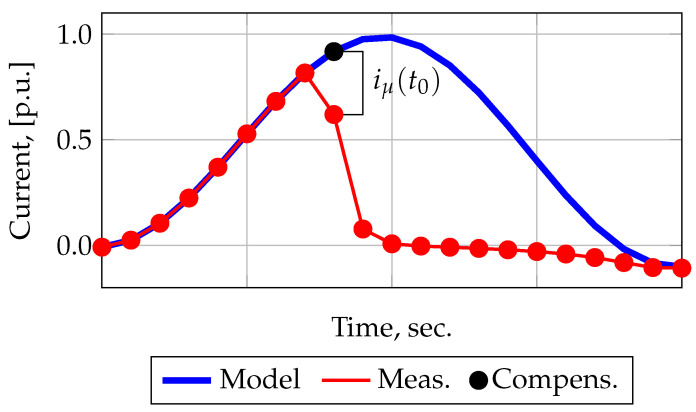
The process of $i_{\mu }$ calculated at the saturation moment $t_{0}$.

In [28,29,30], a method for filtering the distorted measured CT current was proposed. In order to avoid the influence of the initial flux density $B_{0}$ on the result of the A1 method, the authors of [11,12] in [28,29] propose a combination of the A1 method with forecasting methods. This method will further be designated as A5. A more detailed description of the A5 method is offered in [30]. Current filtering by this method is performed using the parameters of both the magnetic and the electrical circuits of the CT. In this method, the procedure for calculating the flux density $B\left(t_{0}\right)$, corresponding to the beginning of saturation, is performed in the opposite direction, i.e., the US is artificially expanded, as a result, for one distorted current sample, one predicted value is obtained. Then, using (12), the magnetizing current corresponding to the beginning of CT core saturation is calculated. This is graphically shown in Figure 3.

(18) $$i_{\mu }\left(t_{0}\right)=i_{2e}\left(t_{0}\right)-i_{2}\left(t_{0}\right),$$

where $t_{0}$ is the the moment of saturation or the end of the US, which corresponds to $B\left(t_{0}\right)$.

Thus, knowing the magnetizing current at the moment of time $t_{0}$, according to Equation (19), it is possible to calculate the magnetic field strength $H\left(t_{0}\right)$, and from the magnetization curve—the flux density $B\left(t_{0}\right)$.

(19) $$H\left(t_{0}\right)=\frac{i_{\mu }\left(t_{0}\right)\cdot w_{2}}{l}$$

Further, knowing $B\left(t_{0}\right)$, using (1) it is possible to calculate the flux density $B\left(t\right)$ corresponding to the saturation interval and then, using (2) and (3), calculate the magnetizing $i_{\mu }\left(t\right)$ and the filtered $i_{1}^{\prime }\left(t\right)$ currents, respectively.

The advantage of the proposed method is better stability with respect to the initial flux density $B_{0}$. However, it should be noted that the first part of the method includes forecasting methods that are highly sensitive to white noise and harmonics. This leads to a decrease in the accuracy of the predicted value of the current $i_{2}\left(t_{0}\right)$, impacting the accuracy of $B\left(t_{0}\right)$. The foregoing can have a significant negative effect on the result of the method used to filter the current measured by class P CTs.

## 3. Testing Current Filtering Methods

### 3.1. Description of CT under Test

In this section, the most effective methods, A1, A2, A3, A4, and A5, have been tested. To carry out approbation, a mathematical model of CT was compiled in the Matlab environment. CT type is TFND-110M (produced in Russian Federation) with closed core. The model uses the following parameters: secondary load $Z_{2}=2.48+j0.2$ Ohm, transformation ratio $n_{T}=600/5$, average magnetic path length l=0.67 m, and cross-sectional area s=17.5 × $10^{-4}$ m^2^. The sampling rate of the model signal was selected in accordance with the IEC 61850 standard—80 points/period. A signal was used as the primary current, the shape of which is described by the Equation (20).

(20) $$i_{1}\left(t\right)=\left\{\begin{array}{cc} 0, & \mathrm{if}\;t<0\\ A\sin\left(100\pi t-\frac{\pi }{2}\right)+Be^{\frac{-t}{0.1}}, & \mathrm{if}\;t>0 \end{array}\right.$$

It is known that the moment of the CT core saturation depends on a number of factors, the main of which are: the initial angle, the amplitude of the periodic component, the decay time constant and the ratio of the short-circuit current, as well as the initial flux density. In this paper, the filtering methods were tested considering changes in the initial flux density and the noise level of the original signal. The following is a description of the conditions and results of simulation experiments.

### 3.2. Results

Simulation Experiment 1.

In the course of this experiment, a current model with parameters corresponding to (20) was set as a reference signal. The initial flux density was assumed $B_{0}=0$ T, white noise was set as δ=0%. The purpose of this simulation experiment is to test the performance of filtration methods A1, A2, A3, A4, and A5.

**Figure 4:**
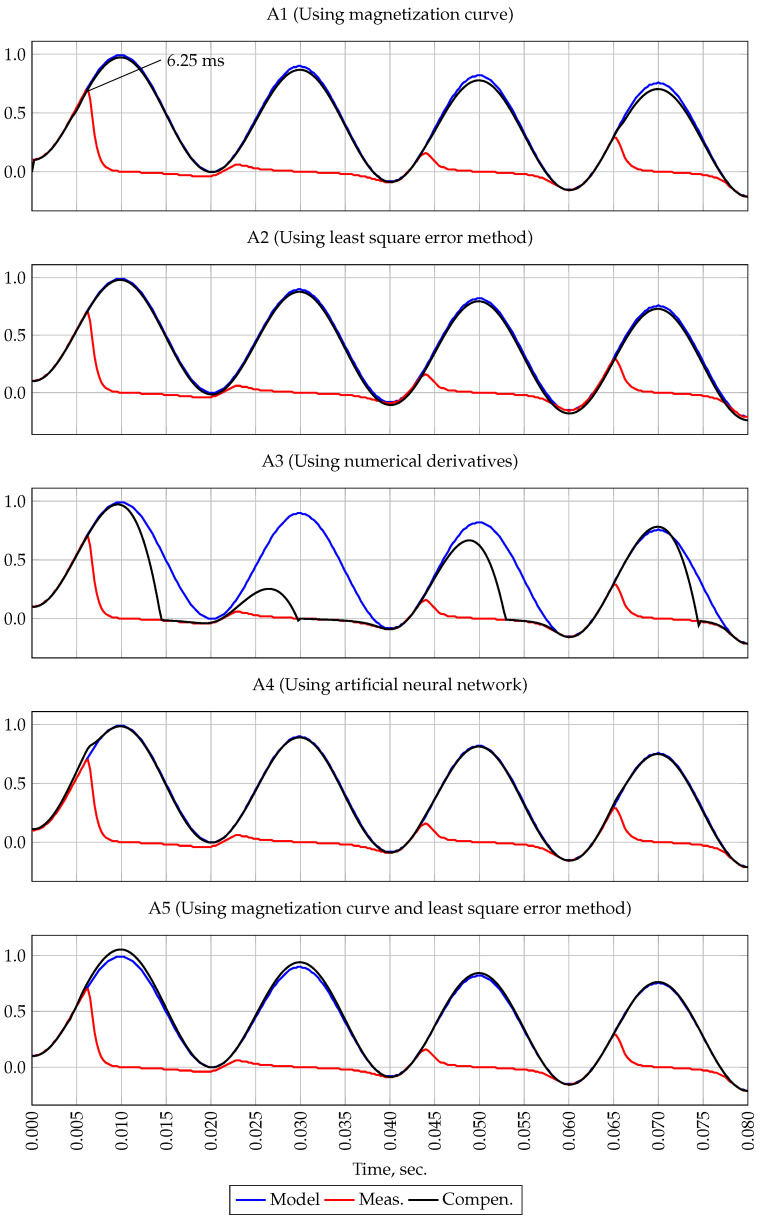
Oscillogram of currents, experiment 1.

Figure 4 shows the reference and measured current signals. As shown in the figure, saturation occurs at time t=6.25 ms.

The first plot of Figure 4 shows the result of the A1 method. Since the initial flux density was not taken into account, the A1 method filters the measured current signal with high accuracy.

The second plot of the Figure 4 shows the result of the first forecasting method A2. When filtering the measured signal for all four periods, the US of only the first interval was included as a measurement at the input of the A2 method, i.e., samples obtained at time t=0–6.25 ms. It can be seen from the figure that this method, in the absence of white noise, provides signal filtering with high accuracy.

The result of the second forecasting method A3 is shown on the third plot of Figure 4. It should be noted that this method is intended to extend the US. However, as shown in Figure 4, with the correct determination of US and in the absence of white noise in the measured current signal, this method can reduce the measurement error when the CT is saturated.

The forth plot of Figure 4 shows the result of filtering by methods using neural networks A4. It should be noted that when filtering the measured signal in the CT saturation mode with the help of neural networks, simulation tools built in the Matlab environment were used. This feature allows one to choose both the topology of neural networks and its learning algorithms. To filter the current signal, the “feedback” topology was chosen, consisting of three levels—input, hidden and output. The network was trained using the Levenberg-Marquardt algorithm with Matlab default parameters. As seen from Figure 4, the A4 method is also capable of filtering the signal with high accuracy.

The result of the combined method A5 testing is shown on the fifth plot of the Figure 4. In the process of filtering, first, using the A2 method, the first predicted value was obtained, then the measured signal was recovered by identifying $B\left(t_{0}\right)$ using the A1 method.

As the results of simulation experiment 1 show, all methods, except A3, are capable of filtering the measured signal received in the CT saturation mode in the absence of white noise and initial flux density.

Simulation experiment 2.

**Figure 5:**
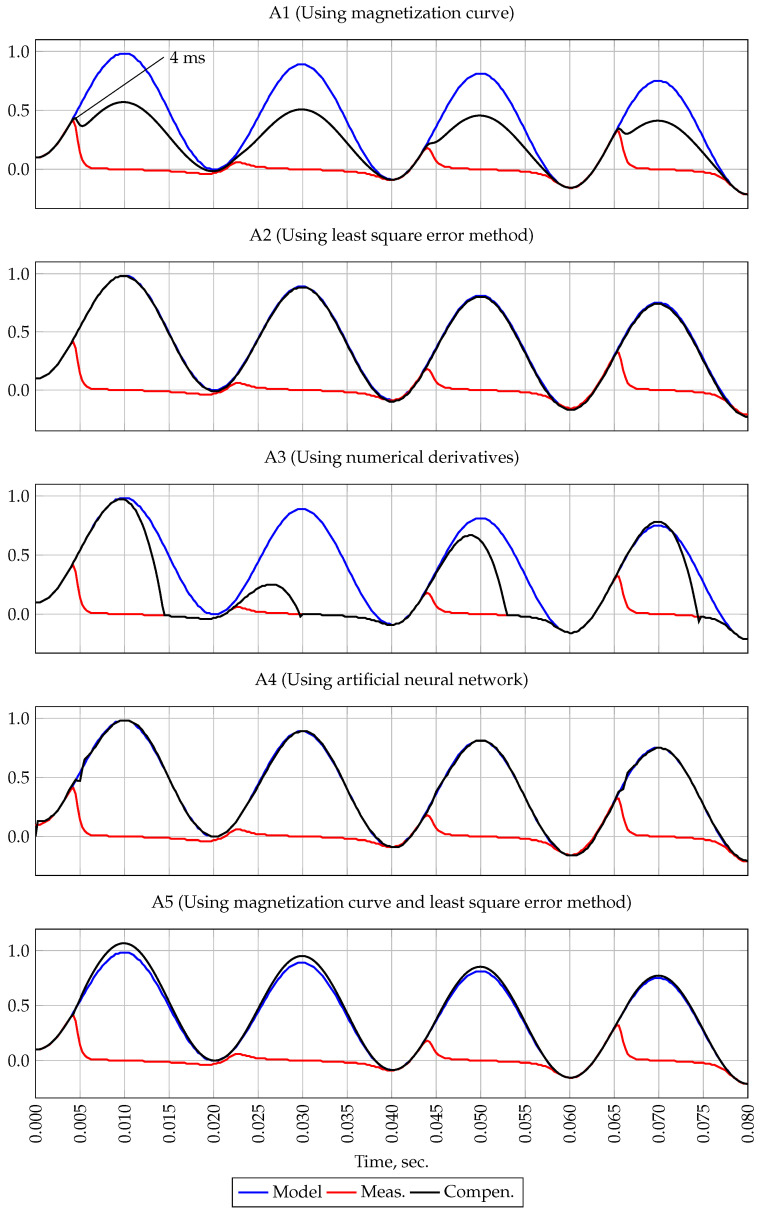
Oscillogram of currents, experiment 2.

The purpose of this experiment is to analyze the effect of the initial flux density $B_{0}$ on the results of the methods. For this, a simulation of the saturation of the CT was performed at $B_{0}=0.05$ T. White noise was specified as δ=0%. The parameters of the current reference signal are identical to those in the previous experiment. Figure 5 shows the reference and measured signals. As shown in this figure, due to the presence of the initial flux density and the coincidence of its sign with the polarity of the reference signal (direct or positive half-cycle), saturation occurs at time t=4 ms.

The result of method A1 approbation is shown on the first plot of the Figure 5. It is clearly seen from this figure that the presence of even a small initial flux density has a significant negative effect on the result of the method.

The second plot of the Figure 5 shows the result of method A2 filtering. As can be seen from this figure, this method has a high stability with respect to the initial flux density and is capable of recovering the measured signal in the presence of a CT $B_{0}$ in the core and in the absence of white noise.

The third plot of the Figure 5 shows the result of method A3 operation. It can be seen that this method also has high stability with respect to the initial flux density $B_{0}$.

The result of the A4 method is shown on the plot of the Figure 5. This method, as well as the A2 method, has a high stability with respect to the initial flux density $B_{0}$.

On the fifth plot of the Figure 5 the result of method A5 is shown. Unlike method A1, this method provides filtering of the measured signal when there is an initial flux density $B_{0}$ in the CT core. Due to the first part of this method—forecasting—the presence of $B_{0}$ did not affect the compensation result.

In the course of the simulation experiment, the methods were tested taking into account the initial flux density at $B_{0}=0.5$ T. The result of the experiment shows that the presence of $B_{0}$ has a significant negative effect on the result of method A1. As for the rest of the methods, they have high stability with respect to $B_{0}$.

Simulation experiment 3.

In the course of this simulation experiment, the stability and sensitivity of the methods with respect to white noise was verified. For this, a reference signal with parameters identical to the previous experiments was set on the primary side of the CT. Then, the measured signal was noised using the Equation (21). Values of $\delta_{1}$ and $\delta_{2}$ in (21) were set equal to 0.03 and 0.01, respectively. The initial flux density was taken $B_{0}=0$ T.

(21) $$i_{meas}\left(t\right)=i_{2}\left(t\right)\cdot \left(1+\delta_{1}\cdot \vartheta \right)+\left(\frac{\max(i_{2})}{n_{T}}\cdot \delta_{2}\cdot \vartheta \right),$$

where $i_{2}\left(t\right)$ is the measured signal; $\delta_{1}$ and $\delta_{2}$ are the a priori specified values of the reference signal noise level based on the assessment of the noise levels in the measurement circuits; ϑ is the random number within the range from −1 to +1 (ϑ has a normal, uniform, distribution), and $n_{T}$ is the CT transformation ratio.

**Figure 6:**
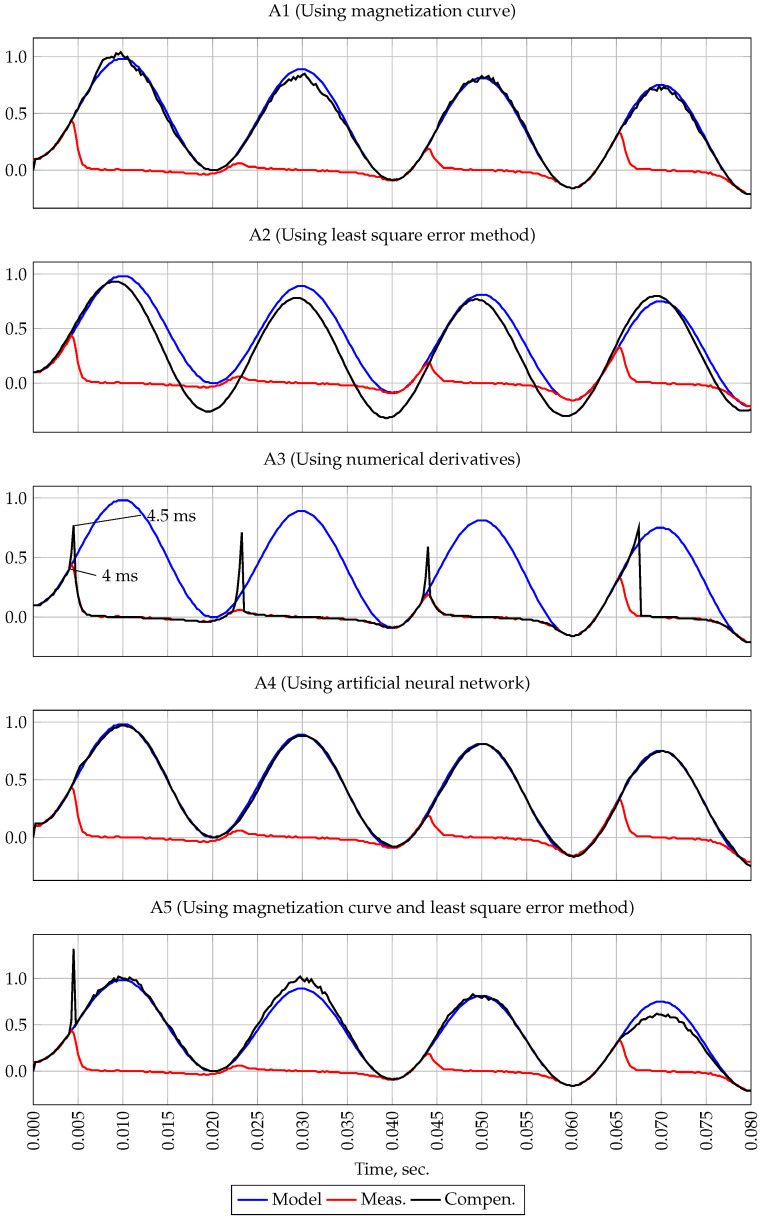
Oscillogram of currents, experiment 3.

Figure 6 shows the reference and measured signal. The moment of saturation in the first period corresponds to 4 ms.

The result of the method A1 approbation with signal noise is shown in the first plot of Figure 6. As can be seen from the figure, this method, in the presence of noise and the absence of an initial flux density, is capable of filtering the current signal with high accuracy.

Figure 6 shows the result of method A2. It should be noted that since white noise implies random number using, a series of calculations (1000 times) were performed to filter the measured signal, for each of which its own filtered signals were obtained. Then, by averaging all these signals, an averaged filtered signal was obtained, shown on the second plot of the Figure 6. It should also be noted that among the filtered signals, signals with a large error in the estimated parameter of the exponential component decay rate were obtained and, as a result, they were dominant when averaged. In order to avoid their influence on the result of the method, they were excluded from the calculation during averaging. However, according to the second plot of the Figure 6 it is still noticeable that method A2 has a high sensitivity to noise.

The result of the method A3 test is shown on the third plot of the Figure 6. As shown in this figure, applying this method in the first half-period, the US was expanded from 4 ms to 4.5 ms. Thus, it was found that the present method has a high sensitivity with respect to white noise and the expansion of the US signal with a sampling rate of 80 points/period is not possible with this method.

The result of filtering the measured signal according to the A4 method is shown on the fourth plot of Figure 6. As can be seen from the figure, the present method is robust against white noise and filters the current signal with high accuracy.

The last plot of the Figure 6 shows the result of A5 method. It is noticeable that this method is capable of filtering a signal with an error acceptable for the correct RPS operation. Small distortions in the second and last periods are caused by the first part of the method—forecasting of the first sample of the measured signal, as well as the second term of the expression (1).

As part of the simulation experiment 3, the methods of filtering the noisy and distorted measured current signal were tested. It was found that in the presence of noise in the measured signal, the error of forecasting methods noticeably increases. The method of magnetization curve, combined methods, and methods with neural networks are able to filter the noisy signal.

Simulation experiment 4.

In the course of this experiment, the filtration methods were tested, taking into account the initial flux density $B_{0}=0.5$ T and white noise with $\delta_{1}=3$% and $\delta_{2}=1$% simultaneously. To reduce the volume of the article, the graphical presentation of the results of this simulation experiment has been omitted. In addition, as in simulation experiment 3, to evaluate the method A2, in the presence of white noise, calculations were carried out, based on the averaging of the results. The results of this simulation experiment are shown below in Table 1 and Table 2.

To compare the methods, the angular, current, and total errors of the filtered signals (in simulation experiments 1, 2, 3, and 4) are calculated. For this, according to (22), the current error expressed as a percentage is determined.

(22) $$f_{i}=\frac{\left|I_{2}-I_{1}^{\prime }\right|}{I_{1}^{\prime }}\cdot 100.$$

The calculation of the total error ε, also expressed as a percentage, is performed using Equation (23).

(23) $$\varepsilon =\frac{100}{I_{1}^{\prime }}\sqrt{\frac{1}{N}\sum_{n=1}^{N}{\left(\frac{i_{1n}}{n_{T}}-i_{2n}\right)}^{2}}.$$

In the above expressions, $I_{1}^{\prime }$ and $I_{2}$ are the RMS values of the primary current and the secondary current, respectively, normalized to the secondary circuit, *N* is the number of samples during one period of the power frequency.

According to IEC 61850, *N* for RPS should be equal to 80 samples/period. It should be noted that the RMS values of the current in (22) were obtained in two ways: by (24) and by the Goertzel algorithm [33]. Further, for convenience, the first method will be designated as M1, and the second as M2.

(24) $$I=\sqrt{\frac{1}{N}\sum_{n=1}^{N}i_{n}^{2}}.$$

According to M2, the phase of the reference, measured, and compensated signals were also calculated. After that, according to (25), the angular error of the methods was estimated.

(25) $$\Delta \varphi =\left|\varphi_{1}-\varphi_{2}\right|,$$

where $\varphi_{1}$ is the reference signal angle and $\varphi_{2}$ is the angle of the measured or compensated signal.

Table 1 shows a comparative analysis of the considered methods with different approaches to filtering the measured current signal. For each, the average and maximum current $f_{i}$ and total ε errors of the filtered signal were determined for the simulation interval—0.08 s. To obtain $f_{i}$ and ε, the effective values of all signals were calculated according to M1. Table 1, the results of those methods are highlighted in bold, the error of which exceeds the maximum permissible value of CT. It should be noted that the maximum current error $f_{i}$ of the measured signal $i_{2}$ in the simulation interval without the use of filtering methods at CT saturation is about 95–96%, and the average is 84–86%.

Table 2 shows the average and maximum current $f_{i}$ and angular δφ errors of the filtering methods over the simulation interval. When calculating $f_{i}$, the RMS values of the signals were obtained using M2. It is worth noting that when determining the phase of a sinusoidal signal, which contains an exponential component, M2 introduces its own error. For example, when evaluating the initial phase of the reference current (14), φ=−90°, the angular error of M2 was 3.6° (4%), when the initial phase was changed by φ=−5°, the error was 0.6° (12%). It should be noted that when determining the RMS value of the signal, M2 does not take into account the exponential component. In the course of simulation experiments, the maximum angular error of the measured signal in the simulation interval varied from 87° to 111°, the average, from 77° to 91°.

### 3.3. Calculation of Methods Errors

**Figure 7:**
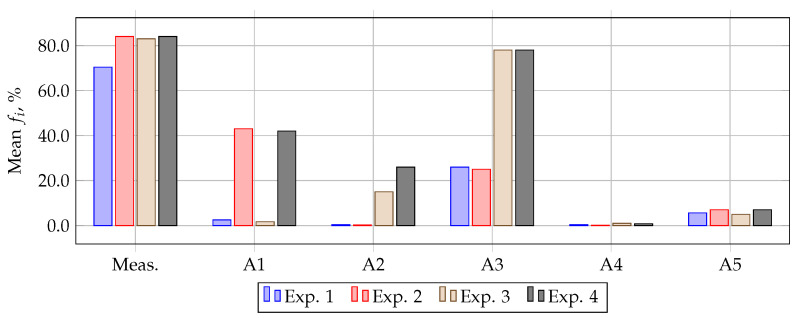
Average current error of filtration methods.

**Figure 8:**
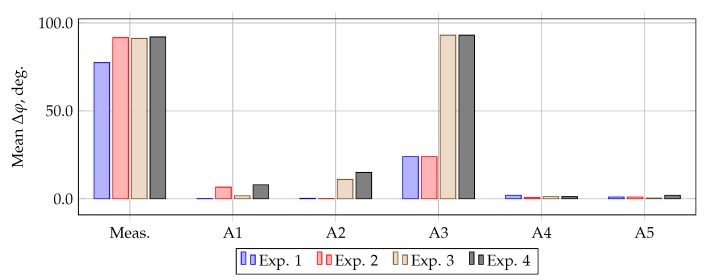
Average angular error of filtration methods.

The average current and angular errors of the measured and compensated currents in the simulation interval are shown in Figure 7 and Figure 8. From Figure 7 it can be seen that with an ideal signal and the absence of magnetization in the CT core, the current error of all methods does not exceed 7.6%. However, in the presence of residual magnetization, the error of method A1 increases sharply, and in the presence of white noise in the measured signal, the error of method A2 increases sharply and exceeds 10%.

From Figure 8 it can be seen that in simulation experiment 1, the largest phase error occurs when using the A4 method. However, in simulation experiment 2, the error of this method decreases, and the error of method A1 increases sharply. In simulation experiment 3, method A2 has the highest error. It should be noted that the A5 method has the highest stability in all simulation experiments.

The results of simulation experiments show that in the absence of white noise in the measured signal and residual magnetization in the CT core, methods A1 and A2 are able to compensate the signal with acceptable accuracy. For example, when evaluated according to M1, their maximum current error $f_{i}$ does not exceed 6.5%, and the total error is 7%, which is acceptable for CTs of accuracy class 10P. When evaluated by M2, the maximum $f_{i}$ was 4%, and the angular error did not exceed 0.7°. However, in the presence of initial flux in the core of the CT, the error of method A1 increases sharply. So, when estimated by M1, the maximum $f_{i}$ is 43%, and by M2, 45%, with a maximum angular error of 12°. The presence of white noise greatly affects the accuracy of methods A2 and A3. For example, in the presence of white noise, the maximum $f_{i}$ of method A2 for M1 is 18%, and for M2 it is 27%, the maximum angular error is 19°. Among the considered methods, the most stable methods proved to be A4 and A5. So, for the most severe mode, in simulation experiment 4, the maximum current error $f_{i}$ of methods A4 and A5 according to Table 1 is 1% and 13%, respectively. According to the Table 2, the maximum $f_{i}$ of these methods is 2% and 10%, and the maximum Δφ is 1.5° and 8°, respectively.

## 4. Discussions

In papers [11,12] in the short circuit mode on the simulation interval (0.18 s) with varying the initial phase, the exponential component of the signal by means of A1, the current signal is filtered, the maximum relative error of which in instantaneous values does not exceed 3% at a sampling rate of 32 points per period.

In [15], a short circuit is imitated, which causes saturation of the CT. The authors, using A2, on the basis of two sections of the US, filter the distorted sections of the current signal over the simulation interval (0.18 s). The results of simulation experiments carried out, by varying the secondary load of the CT, the decay time constant of the exponential component. The value and the initial phase of the short-circuit current show that in 95% of cases the distorted sections of the current signal can be filtered by the A2 forecasting method, the maximum current error of which does not exceed 14.94%.

In [19], by varying the magnitude and the initial phase of the short-circuit current, the time constant of the exponential component, the secondary load and the initial flux of the CT, the CT saturation is simulated. Then, using A4, the measured current signal is filtered. It is shown that when filtering the measured current signal, the maximum current error in the simulation interval (0.2 s) does not exceed 0.77%. In [22], simulation experiments are performed by varying the above factors over a simulation interval of 25 ms, where the maximum current error does not exceed 2.52%.

In [28], the distorted current is filtered based on the A5 method. The simulation of the short circuit causing saturation is performed by varying the initial phase, initial flux, and constant time of the exponential component. In [28], the accuracy of the method is estimated by calculating the relative error of the filtered current from instantaneous values. Thus, the maximum relative error of the filtered current signal in the simulation interval (0.12 s) does not exceed 1.5%.

For a numerical analysis of the filtering accuracy of the current signal, the results of the above methods can be compared with the current errors given in Table 2 and Table 3. For clarity of the methods considered in this paper, properties, advantages, and disadvantages, as well as the approaches used to filter the signal are given in the table form below.

To improve the current filtering in the future, it is proposed to improve the combined method in terms of determining the initial flux by iterative methods. For its practical application, it is proposed to develop detection methods on the basis of searching the stable sections of the flux density corresponding to the saturation mode, as well as on the basis of identifying the deviation of the measured current from the sinusoidal one.

## 5. Conclusions

In this paper, a comparative analysis of filtering the measured CT current in the saturation mode methods is carried out. The elaboration degree on topic of increasing the accuracy and reliability of measuring information at the CT saturation is revealed. Methods of US selection are considered, their advantages and disadvantages are described. A comparison of filtration methods stability and sensitivity for white noise in the measured current and initial flux of the CT is carried out. It should be noted that when testing filtration methods, to simplify calculations, the selection problem, which is a key part of the compensation of the CT error in the saturation mode, was considered complete, although in real conditions the application of filtration methods without solving the selection problem is not possible. It should also be noted that the most promising method is the combined A5. However, for its practical application, it is necessary to improve the accuracy and reliability of determining the initial flux of CTs. 

## Figures and Tables

**Table 1:** Comparison of signal filtering methods in the saturation mode of current transformers when determining the effective value of signals according to M1.

Method and Reference	Computational Experiment 1				Computational Experiment 2				Computational Experiment 3				Computational Experiment 4			
	$f_{i}$, %		ε, %		$f_{i}$, %		ε, %		$f_{i}$, %		ε, %		$f_{i}$, %		ε, %	
	Max	Avg.	Max	Avg.	Max	Avg.	Max	Avg.	Max	Avg.	Max	Avg.	Max	Avg.	Max	Avg.
A1 [11,12]	6.5	4	7	4.2	43	42	44	43	7.2	3.4	8	5	49	43	52	44
A2 [15,16,17]	4	3	6	4	2	1.2	3	2	18	10	38	32	23	12	65	59
A3 [18] *	79	37	90	58	79	36.6	90	58	91	75	100	98	91	75	100	98
A4 [19,20,21,22,23,24,25]	1.2	1	4	2	0.3	0.2	3	2	1.44	1	3.2	2.8	1	0.3	3	2
A5 [28,29,30]	6.2	3.6	6	4	8	5	8	6	6	4	9	5	13	8.6	27	11

* this method is intended to extend the US.

**Table 2:** Comparison of signal filtering methods in the saturation mode of current transformers when determining the angle and the effective value of signals according to M2.

Method and Reference	Computational Experiment 1				Computational Experiment 22				Computational Experiment 3				Computational Experiment 4			
	$f_{i}$, %		Δφ, °		$f_{i}$, %		Δφ, °		$f_{i}$, %		Δφ, °		$f_{i}$, %		Δφ, °	
	Max	Avg.	Max	Avg.	Max	Avg.	Max	Avg.	Max	Avg.	Max	Avg.	Max	Avg.	Max	Avg.
A1 [11,12]	3.7	2.5	0.7	0.2	45	43	12	6.6	4.3	1.7	3	1.7	43	42	14	8
A2 [15,16,17]	1	0.4	0.7	0.3	0.7	0.3	0.4	0.16	27	15	19	11	48	26	27	15
A3 [18]	37	26	30	24	37	25	30	24	84	78	109	93	84	78	110	93
A4 [19,20,21,22,23,24,25]	2	0.4	3.5	2	1	0.2	1	0.7	2	1	1.6	1.3	2	0.8	1.5	1.2
A5 [28,29,30]	7.6	5.6	2	1	9	7	2	1	6.5	5	1.3	0.4	10	7	8	2

**Table 3:** Advantages and disadvantages of filtration methods.

Method and Reference	Approach	Advantages and Disadvantages
A1 [11,12]	Based on the use of the magnetization curve	(+) High stability with respect to white noise, the ability to filter a signal regardless of US. (−) High sensitivity to initial flux density.
A2 [15,16,17]	Based on the use of US samples	(+) No dependence on CT parameters and high stability with respect to the initial flux density. (−) High sensitivity relative to white noise, US referenced.
A3 [18]		
A4 [19,20,21,22,23,24,25]	Based on the use of neural networks	(+) High accuracy in the presence of both initial flux density and white noise, there is no dependence on US and CT parameters. (−) To take into account all the factors affecting the occurrence of saturation, a large amount of memory of the microprocessor device is required. It is also necessary to solve a number of problems related to the accuracy of the current saturation mode recognition by the neural network.
A5 [28,29,30]	Based on the use of the magnetization curve and ICT samples	(+) Stability with respect to the initial magnetic induction and white noise. (−) Dependence on the parameters of the CT magnetic circuit and the number of measured signal in the IPT samples. The accuracy of the method depends on the forecasting methods used.

## Data Availability

Not applicable.
